# Weaning from Mechanical Ventilation in ARDS: Aspects to Think about for Better Understanding, Evaluation, and Management

**DOI:** 10.1155/2018/5423639

**Published:** 2018-10-09

**Authors:** Iuri Christmann Wawrzeniak, Silvia Regina Rios Vieira, Josué Almeida Victorino

**Affiliations:** ^1^Programa de Pós-Graduação em Ciências Médicas, Universidade Federal do Rio Grande do Sul, Porto Alegre, Brazil; ^2^Hospital de Clínicas de Porto Alegre, Brazil; ^3^Universidade Federal de Ciências da Saúde de Porto Alegre, Brazil

## Abstract

Acute respiratory distress syndrome (ARDS) is characterized by severe inflammatory response and hypoxemia. The use of mechanical ventilation (MV) for correction of gas exchange can cause worsening of this inflammatory response, called “ventilator-induced lung injury” (VILI). The process of withdrawing mechanical ventilation, referred to as weaning from MV, may cause worsening of lung injury by spontaneous ventilation. Currently, there are few specific studies in patients with ARDS. Herein, we reviewed the main aspects of spontaneous ventilation and also discussed potential methods to predict the failure of weaning in this patient category. We also reviewed new treatments (modes of mechanical ventilation, neuromuscular blocker use, and extracorporeal membrane oxygenation) that could be considered in weaning ARDS patients from MV.

## 1. Introduction

Since the creation of the intensive care unit (ICU), the use of mechanical ventilation (MV) has been evaluated basically with regard to three aspects of debate [1]. The first is when should MV be started, either invasive or noninvasive. The second is after the start of MV, when the choice of ventilation mode is made and ventilation parameter settings are evaluated. These adjustments were better understood after the description of “ventilator-induced lung injury” (VILI), where adjustments made only for the correction of gas exchange may worsen pulmonary and extrapulmonary injury [2–4].

The third subject of debate, which is no less challenging, is when would be the best time for withdrawal of ventilatory support, so-called weaning from MV [5]. MV weaning has been studied for several years and has gone from a state of the art to a science after formulating more defined concepts and conducting clinical studies. MV weaning can be simple in most cases, but there may be cases of difficult or prolonged weaning. In these groups, the outcomes are worse when compared to simple weaning from MV [6]. This more complicated MV weaning scenario has been seen more in the last years after the best initial care of the critical patient, which has provided a reduction in mortality, but a portion of patients progress to chronic critical illness [7, 8].

Studies and guidelines for MV weaning have little concern for distinguishing the peculiarities of the critical patient [9–13]. There is no individualization of a patient with chronic obstructive pulmonary disease (COPD) or acute respiratory distress syndrome (ARDS). The first shows the changes of a chronic lung disease, while the second displays various acute peculiarities related to intense inflammatory response. New approaches could be considered in MV weaning with the evolution of intensive care [14].

This study proposes a review of the main aspects for the understanding of ARDS during weaning of MV as well as evaluating and managing this phase of withdrawal of ventilatory support. The review of the literature was carried out aiming at the aspects related to the evaluation of weaning of MV in patients with ARDS. Search was conducted in the period from 1967 to 2018 and the following electronic databases: MEDLINE, EMBASE, LILACS, and Cochrane Central Register of Controlled Trials (CENTRAL) and relevant science sites. The following “Mesh terms” are from MEDLINE: “Respiratory Distress Syndrome, Adult”[Mesh], “Ventilator Weaning”[Mesh]. After the results, the articles of relevance to the theme of the proposed study were selected.

## 2. Mechanical Ventilation in ARDS: Start, Transition, and End of Weaning Process

The initial evaluation and treatment of the patient with ARDS begin with the correction of the inflammatory mechanism that triggered the process, i.e., sepsis and the decision to start ventilatory support [17, 18]. This ventilatory support can be provided as supplemental oxygen, high-flow nasal oxygen therapy, and noninvasive MV and in most cases invasive MV [19–21]. Another important initial aspect of ventilatory support is the choice of ventilatory mode (controlled versus spontaneous) and parameter settings to adopt the “lung-protective ventilation” strategy. This protective ventilation consists in the adjustment of low tidal volume (TV) on the basis of predicted weight and elevated levels of positive end-expiratory pressure (PEEP) with respiratory regulation (RR), considering not only the correction of hypoxemia, but in the care of the targets of pulmonary pressures and volumes to avoid volutrauma and atelectrauma [22–24]. In the most severe cases, the use of neuromuscular blockade (NMB), prone position, and extracorporeal membrane oxygenation (ECMO) will be evaluated when there is refractory hypoxemia [25–29]. These initial strategies are key to successful treatment of patients with ARDS and therefore weaning success and reduction in MV time.

**Figure 1:**
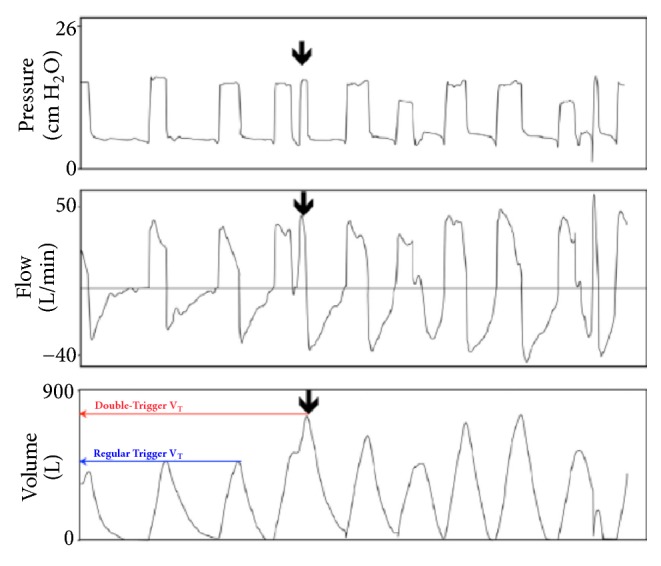
Double‐triggering occurs when a spontaneous effort triggers a (second) ventilator breath before the initial breath has completely exhaled (arrow). The pressure-time trace (upper panel) and flow-time trace (mid panel) demonstrate the occurrence of the additional breath, but do not give a sense that both inspirations are summed; this is apparent from the volume-time (lower panel) trace indicating that the double-triggering results in a substantially larger (potentially injurious) VT (red) compared with regular triggering (blue). Legend: VT: tidal volume. With authors permission [15].

**Figure 2:**
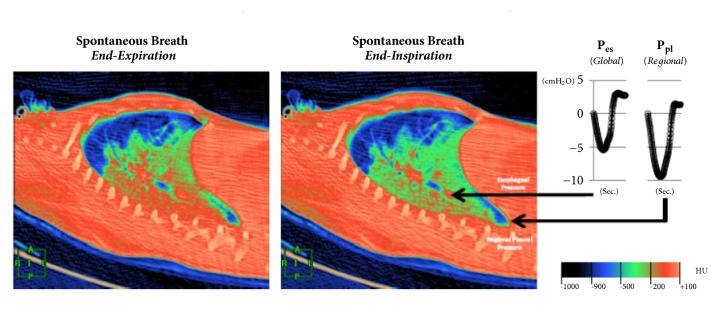
Dynamic CT scan in end-expiration (left panel) demonstrates that the aerated lung (blue) is nondependent, while the dependent lung is densely atelectatic (red). At end-inspiration during a spontaneous breath (mid panel), there is little change in the nondependent aerated lung (blue); the dependent lung, previously densely atelectatic (red) is now partially aerated (green/red). The inspiratory pleural pressure traces (right panel), measured at the arrow tips, show the negative deflections (“swings”) in regional Ppl and global Pes during inspiration. However, the “swing” in regional Ppl is greater (x2) than the “swing” in Pes, indicating that diaphragm contraction results in greater distending pressure applied to the regional lung near the diaphragm compared with the pressure transmitted to the remainder of the lung (i.e., Pes). Ppl: pleural pressure; Pes: esophageal pressure; HU: Hounsfield Units, with authors permission [15].

**Figure 3:**
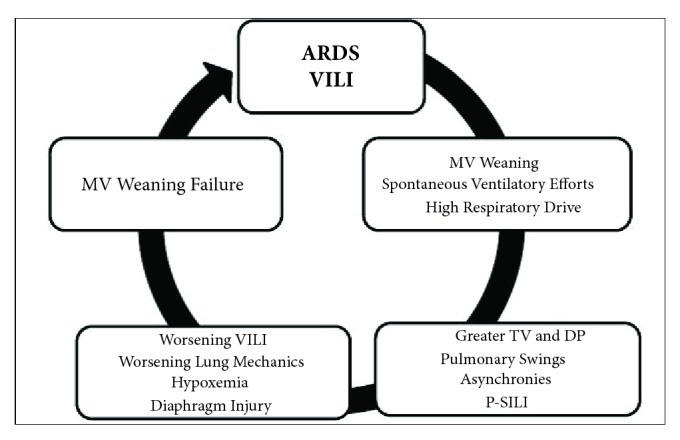
Weaning failure in ARDS. ARDS: “Acute Respiratory Distress Syndrome”; VILI: “Ventilator-Induced Lung Injury”; MV: “Mechanical Ventilation”; TV: “Tidal Volume”; DP: “Driving Pressure”; P-SILI: “patient self-inflicted lung injury”.

After a few days with assumed improvement in inflammatory status and gas exchange, the clinician at bedside begins to think of withdrawing ventilatory support. First, NMB and sedation are provided until spontaneous ventilation movements are detected. Afterwards, spontaneous breathing trials (SBTs) are conducted to evaluate the withdrawal of MV. The T tube test or pressure support ventilation (PSV) modalities are useful for all types of MV weaning patients [12]. In recent years, the influence of spontaneous ventilation has been better evaluated in patients with ARDS. Initial studies suggest the beneficial effect of spontaneous ventilation in both improvement of hypoxemia and pulmonary compliance and reduction in atrophy of respiratory muscles, mainly diaphragmatic [30–33]. However, animal studies show the opposite with increased transpulmonary pressure, worsening of asynchrony (flow starvation, short cycling, and double-triggering), breath stacking, pendelluft phenomenon, diaphragmatic injury, and worsening of inflammatory response and VILI (Figures 1 and 2) [15, 16, 34–37]. Spontaneous efforts may cause swings and heart-lung imbalances with worsening of pulmonary edema and injury, mainly due to excessive negative pleural pressure (Ppl) [38, 39]. During weaning, excessive respiratory drive and high ventilatory demands increase dyspnea and may lead to weaning failure and/or failure to intubate and may present “air hunger” [40]. The high respiratory drive leads to vigorous inspiratory efforts that result in excessive global or regional pulmonary distension due to a nonhomogeneous distribution of stress and strain. A mechanism recently termed “patient self-inflicted lung injury” (P-SILI) may create a vicious circle of worsening injury, resulting in higher TVs and injurious lung stress [19]. Papazian et al. showed a reduction in mortality and inflammation in moderate and severe ARDS with the use of NMB in the early stages, suggesting the attenuation of lung injury with NMB [25, 41]. These findings related to the presence of spontaneous movements in injured lungs should be considered in patients with ARDS, who are starting weaning from MV. Despite the great interest in this area, there are few studies that definitively assess the true impact of spontaneous ventilation during weaning from MV in patients with ARDS (Figure 3).

## 3. Monitoring MV Weaning in ARDS

The usual parameters for the evaluation of MV weaning are in regard to clinical, gasometry, ventilatory mechanics, and radiological data. These parameters can assess the overall improvement in the cause of respiratory failure. However, they may be unable to predict patients with MV weaning failure.

The use of frequency-to-tidal volume ratio (f/VT) is the most widespread predictor of weaning and has better prediction than other prediction methods. However, the f/VT as well as the other methods have failures to predict the withdrawal of the MV. New applications of weaning predictors in this scenario must be explored in future studies [65]. During the weaning of ARDS patients in PSV, it was observed in a pilot study in ARDS patients with weaning failure that TV increases without changes in RR patients as generally described by Tobin [5, 58]. These alterations—reduced TV and increased RR—associated with other signs of failure—arterial hypertension, sweating, accessory musculature utilization, and drowsiness—usually occur in later phases of MV weaning failure.

The evaluation of hypoxemia is generally used to classify the severity of ARDS and monitor the progression of lung injury [66, 67]. At weaning from MV, usually a PaO_2_/FiO_2_ ratio over 200 is considered the criterion for the start of the MV weaning process [13]. Nevertheless, hypoxemia is not a specific marker of the inflammatory response [68].

Another point to emphasize is that, during spontaneous ventilation tests, there is the inability to evaluate transpulmonary pressure or driving pressure (DP) [56, 69]. During spontaneous ventilation after withdrawal of NMB, other pressures involved—pleural and muscular pressure—influence the evaluation of pulmonary mechanics [70]. Pilot study for early weaning evaluation was seen to be increased DP. For the evaluation of pulmonary mechanics during study, small doses of sedatives or short-acting NMBs (propofol 10mg IV and if necessary succinylcholine 2-4mg IV) may be given [58].

The evaluations of airway pressures usually employed in MV weaning assessment are the levels of PEEP and peak and plateau pressures. The target generally recommended to start weaning from MV is a PEEP level of 5-8 cmH_2_O and pressure support levels to maintain adequate ventilation.

In patients with ARDS there is a wide variation of radiological findings seen on conventional chest radiography compared with computed tomography [71]. There is also a discrepancy of the observers in the interpretation of the presence of edema seen in the chest radiograph [18].

## 4. Potential Methods for Predicting MV Weaning in ARDS

The purpose of this topic is to present some methods that have been the subject of debate in the literature and that could be better evaluated for weaning prediction in ARDS patients (Table 1). However, the magnitude of weaning from MV specifically in patients with lung injury as well as new methods should be further studied. However, clinicians need new parameters at bedside to better predict MV weaning because of the unique pathophysiology of patients with ARDS compared to other causes of respiratory failure.

### 4.1. Monitoring Mechanical Ventilation Parameters

The monitoring of pulmonary pressures through the usual pressure curves shown by ventilators during weaning is mixed by the presence of spontaneous ventilation. The pressures abolished by the effect of the BNM appear after their withdrawal and can influence the lung lesion as well as during the transition from controlled to spontaneous [70, 72]. Amato et al. in a large retrospective analysis showed the increase in DP as a worse predictor of outcome in patients with ARDS [56]. At weaning from MV, the persistence of the inflammatory response could increase DP. This alteration could be better evaluated as a new marker of complicated weaning in lungs that still had unresolved “occult” lung injury.

The use of esophageal manometry has been used in respiratory physiology research, but its clinical use is not common. The evaluation of esophageal pressure (Pes) using esophageal manometry helps in the estimation of pleural pressures because its measurement can be influenced by the effects of the chest wall and lungs [42]. There is a gradual reduction in ventilatory support and increased patient effort during weaning from MV. The Pes increases progressively in patients who fail weaning, while the success of weaning does not occur significant changes in Pes [73, 74]. The continuous evaluation of Pes variations predicts a better success or failure at MV weaning than f/TV [74]. Yoshida et al. suggest the use of the esophageal manometry to guide PEEP settings to reduce VILI [57]. Pes monitoring may be a useful tool and part of the intensivist's clinical armamentarium to show oscillations of pulmonary pressures during weaning from MV [42, 43].

The pressure developed in the occluded airway 100 ms after the onset of an inspiratory effort (P0.1) is another measure that could help as a weaning predictor in patients with ARDS. P0.1 was initially described more than 40 years ago [75] and may be used to assess simply and noninvasively the increased ventilatory drive in ARDS and its deleterious consequences in injured lungs [40]. P0.1 is independent of respiratory mechanics and the patient's reaction, and it is, importantly, unaffected by respiratory muscle weakness. The optimal target range for respiratory drive and inspiratory effort during MV is uncertain. In healthy subjects breathing at rest, P0.1 varies between 0.5 and 1.5 cmH_2_O [44]. P0.1 can be useful to adjust the level of ventilatory support due to its close correlation with inspiratory effort. Higher values of P0.1 indicate insufficient levels of support, while lower values correspond to excessive assistance. P0.1 has been extensively studied as a predictor of weaning success or failure [5]. A high P0.1 during a spontaneous breathing trial is associated with failure, suggesting that a high respiratory drive could predict weaning failure. P0.1 alone can provide clinicians with information regarding their patient's drive, where it is sensitive to ventilator settings and may be useful during weaning [45].

### 4.2. Imaging Monitoring

Lung ultrasound can be a good alternative to chest radiography or computed tomography scan in many cases. Bedside lung ultrasound in the evaluation of patients with respiratory failure has been well established [76, 77]. In ARDS there are several findings in the pulmonary ultrasound [71]. The evaluation of aeration can predict the success or failure of weaning from MV [46–48]. Bouhemad showed to be accurate the use of the lung ultrasound reaeration score for the use of antibiotics in ventilator-associated pneumonia [60]. Haji et al. showed parameters lung (loss of aeration score of the left and right anterior and lateral regions) and parameters cardiac through diastolic dysfunction (left atrial area, E/E′, interatrial septal rightward fixed curvature) to help predict failed extubation [49]. Echocardiography should be further explored in this population because the risk of swings and changes in heart-lung interaction can influence the success of weaning from MV [38, 78]. The lung ultrasound examination has some limitations: it cannot detect lung overinflation; subcutaneous emphysema and the presence of large thoracic dressings may preclude propagation of ultrasound beams to the lung surface, with severe chest trauma or burns; it may be limited by the patient's pain and discomfort; training is required to correctly perform the examination and interpret its findings and it is not a continuous monitoring tool [71]. Lung ultrasound and echocardiography are still uncertain methods in the evaluation of weaning in ARDS and require more specific studies for their present application.

**Figure 4:**
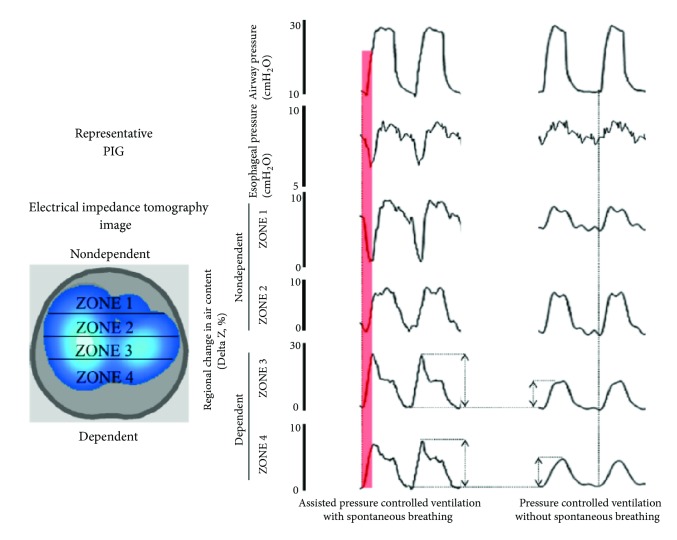
Electrical impedance tomography (EIT) waveforms in experimental lung injury, spontaneous versus ventilator breaths. In an anesthetized pig model of acute lung injury assist pressure-controlled ventilation (IP, 15 cm H2O; f, 25 min21; PEEP=13 cm H2O; triggering threshold, 22 cm H2O) was used. The EIT image was divided into four zones, each covering 25% of the ventrodorsal diameter (zones 1–4). During controlled ventilation (under muscle paralysis), simultaneous inflation of each of the different lung regions was observed, although at different inflation rates. In contrast, when spontaneous efforts were present, two observations were noted. First, in the initial stages of the breath, spontaneous efforts caused inflation of dependent lung regions (red in zones 3 and 4), which was greater with controlled breaths. Second, the early inflation in the dependent region was accompanied by concomitant (transient) deflation of nondependent region (red in zone 1), indicating movement of gas from nondependent to dependent lung regions, because this was not associated with alterations in tidal volume it indicates a pendelluft phenomenon. This finding was always present during spontaneous breathing efforts in all animals with experimental lung injury: f = respiratory frequency; IP: inspiratory pressure; PEEP: positive end-expiratory pressure, with authors permission [16].

**Figure 5:**
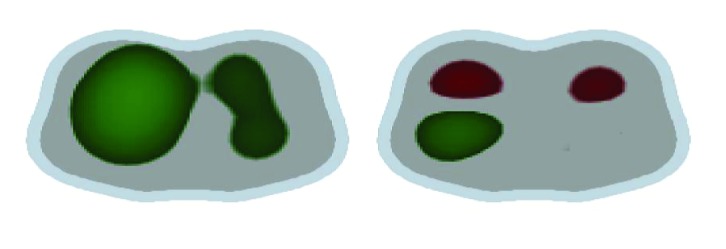
Electrical Impedance Tomography. Example of the visualization of the variation in pulmonary ventilation seen through EIT of MV weaning in two ARDS patients in the first 2 hours. Image on the left indicates the gain in ventilation in green with an increase in TV from 6 to 12 ml/kg. The patient on the right showed weaning failure and prolonged weaning from MV. Image displays an increase in TV from 6 to 8 ml/kg and loss of ventilation variation in red; the patient showed simple weaning from MV.

Another imaging tool to use at bedside is electrical impedance tomography (EIT), which is a noninvasive imaging method [79]. EIT is a radiation-free, noninvasive technique for continuous monitoring of lung volume during ventilation and possibly a guide for the weaning process [51, 52, 80]. The dynamic real-time evaluation of aerated and nonaerated areas could show the pulmonary swings and their correlation with the course of MV weaning (see Figure 2). Blankman et al. compared the effects of pressure control ventilation (PCV) and PSV on the distribution of ventilation with the use of EIT. There was improved ventilation of the dependent lung region during PSV due to the contribution of the diaphragm resulting in a distribution shifted to the nondependent lung with elevated TV [81]. Bickenbach et al. showed changes in regional ventilation of the lung and heterogeneity in prolonged weaning undergoing T-piece trials in real time [53]. Regional EIT monitoring during edema formation reveals a decrease in lung aeration in dorsal regions, associated with a decrease in regional ventilation. In association with such changes, EIT typically discloses compensatory increases in regional ventilation of ventral regions. The presence of spontaneous ventilatory movements causes increased ventilation in dorsal regions. This ventilation is an effect of apposition of the dorsal diaphragm and also gravitational effect, leading the dependent lung to a greater regional complacency [54]. In contrast, high pressure support levels or TVs are associated with increased ventral ventilation and signals of nondependent lung overdistension (Figures 4 and 5). When pendelluft occurs, the possibility of overstretch of the dependent lung is strongly suggested by EIT, even in patients subjected to low TV ventilation [82]. Impedance properties are sensitive to the difference between blood and air; therefore, EIT has also been studied to assess the regional distribution of perfusion and its relationship with ventilation. The lung pulsatility method has so far been shown to provide qualitative information about lung perfusion, e.g., following the activation of hypoxic pulmonary vasoconstriction [83].

Another evaluation during weaning from MV is the use of plethysmography, a tool to assess functional residual capacity, TV, and variability over time. Studies with pulmonary variability have correlated with success or failure of MV weaning in patients in general [84–86]. Studies have been carried out recently with the use of EIT, but there is still a need to improve image reconstruction and to create algorithms for applications in weaning evaluation at bedside [87].

### 4.3. Monitoring Asynchrony

Ventilator asynchrony is associated with increased ICU stay and mortality. During episodes of asynchrony may occur worsening hypoxemia and increased respiratory muscle work [50, 59, 88]. There is also increasing concern that asynchrony may cause large transpulmonary pressure swings and inappropriately large TV that may be especially harmful in critically ill patients who are receiving lung-protective ventilation [36]. The presence of asynchrony during the weaning of ARDS patients from MV could be better quantified and distinguished. In addition, the types of asynchrony could also be evaluated. The presence asynchrony, both quantity and type, could be considered at bedside as a predictor MV weaning failure in patients with ARDS.

### 4.4. Biomarkers

ARDS is characterized by intense inflammatory response with release of several inflammatory mediators during the course of this response, exudative, proliferative, and fibrotic phases of ARDS [18]. Increased levels of plasma biomarkers, including markers of systemic inflammation (interleukin-6 and interleukin-8), epithelial and endothelial injury, along with markers of dysregulated coagulation, have been associated with adverse outcomes of ARDS [18, 89, 90]. During the weaning process, SBTs involve cardiopulmonary stress for ventilated patients; interleukin-6, a major modulator of the stress response, has been shown to be higher in COPD patients during weaning failure [91]. Yang et al. showed reduced levels of serum inflammatory cytokines, especially IL-6, with successful weaning in septic patients on ventilators [55]. Other more specific lung biomarkers could also be evaluated during weaning from MV in patients with ARDS, i.e., amphiregulin and type III procollagen [92, 93]. However, there are still no studies that define the role of biomarker measurement in the evaluation of MV weaning in ARDS patients.

## 5. Innovative Therapies to Treat ARDS with Complicated Weaning

There have been few studies specifically on the subject of weaning ARDS patients from MV and on their approach as well [94, 95].  Table 2 has future potential suggestions to resume the actual moment in research that is waiting research to confirm this evaluation in VM weaning for ARDS. Weaning that does not progress should be evaluated for any factors that perpetuate the inflammatory response, e.g., uncontrolled infection. Invasive ventilation itself can lead to iatrogenic damage to the lungs already with lung injury, and therefore, caution with the influence of spontaneous ventilation can lead to more lung injury and diaphragmatic dysfunction. New occult mechanisms increasing the risk of VILI during assisted spontaneous breathing (e.g., occult pendelluft and solid-like lung behavior) are extremely difficult to recognize at bedside, but clinically, they should be suspected in patients with more severe lung injury (e.g., patients with extremely low compliance) and/or with strenuous inspiratory effort [72].

### 5.1. New Ventilatory Modes

The ventilation mode should be evaluated to improve patient-ventilator synchrony. When pressure support is added to spontaneous breathing, the same principles apply, but total pressure across the respiratory system and transpulmonary pressure increase, generating additional flow and volume. During pressure support, if inspiratory airflow exists after the end of respiratory muscular pressure, Pes/Ppl can result in positive swings along inspiration, where ventilation is a hybrid of active (during the first part) and passive (towards the end) phenomena [70].

An alternative ventilatory mode that the ventilator generates pressure in proportion to the patient's effort is the proportional-assist ventilation (PAV) mode. The synchrony can improve because the RR is determined by the patient's own respiratory drive of the patient and the ventilatory assistance terminates with the end of the inspiratory effort. The different approaches used in PSV and PAV to pressurize the lung could theoretically lead to marked differences in response to these variations in respiratory system impedance. Respiratory loading has often been used to simulate changes in respiratory impedance and to evaluate the consequences of such changes on ventilatory patterns and respiratory muscle performance [61].

Another ventilatory mode that is adjusted to the neural output of the patient's respiratory center is neurally adjusted ventilator assist (NAVA). The pressure is regulated by the integral of the electrical activity of the diaphragm (EAdi) and therefore better synchrony between the patient and the ventilator. Studies show that NAVA protects against excess pressure and TV when compared to PSV. The Hering-Breuer reflex may be implicated in the absence of a TV increase with NAVA levels [96]. Terzi et al. evaluated ARDS patients and showed recovery and improved the synchrony compared PSV [62]. Therefore, NAVA is another ventilation mode that improves patient-ventilator synchrony in these patients during the weaning process [63, 97–99].

### 5.2. Partial Neuromuscular Blockade

An alternative but still experimental approach with “partial neuromuscular blockade” proposed by Doorduin et al. in 10 patients with ARDS during PSV is innovative and deserves attention [100]. This study showed a reduction in TV, EAdi, and transpulmonary pressure with subtherapeutic doses of rocuronium without changes in pH and diaphragm activity. In other words, strong spontaneous breathing efforts were abolished, but a significant degree of diaphragm activity was maintained. An assist-controlled mode under these conditions resulted in severe breath stacking, which is associated with high TV [31]. A change of mode of ventilation to controlled ventilation and adjustment of sedatives (propofol or dexmedetomidine, for example) could help in controlling the high respiratory drive.

### 5.3. Extracorporeal Membrane Oxygenation

Another device that has gained ground in intensive therapy is ECMO. After a series of cases with H1N1 and the CESAR study, its use has been more widespread and studied [27]. Authors suggest that ultra-protective MV can be better performed with this device especially in patients with spontaneous ventilatory movements and control of ventilatory drive in patients with ARDS [64, 101–104]. Its use in patients with difficulty in weaning needs to be better studied because there is physiological and protective rationale as a whole. However, the risks of the procedure and the absence of robust studies in this situation do not allow its routine use in weaning patients with ARDS.

## 6. Conclusion

Weaning ARDS patients from MV still needs to be better debated and studied. New knowledge related to the presence of spontaneous ventilation and the risk of inflammatory worsening are important in this debate. Practitioners could consider weaning in ARDS to continue to protect the lung. New methods to evaluation of weaning of the patients with ARDS as well as more rational approaches based on pathophysiology should be performed for success in withdrawal of ventilatory support and improvement of their outcomes.

## Figures and Tables

**Table 1:** Potential methods for predicting weaning in ARDS.

Potential Method	Advantages	Disadvantages
Esophageal pressure[42, 43]	Pressure measurements with spontaneous ventilation Quantification of pulmonary swings Help in the visualization of asynchrony	Difficulty in positioning the catheter and its accuracy for measuring esophageal pressure Minimally Invasive

P0.1[44, 45]	Evaluation of ventilatory drive	Failure of previous studies as a predictor of general weaning failure

Lung ultrasound [46–48]	Non-invasive Quantification of aeration and collapse during weaning	Operator dependent Skin lesions may make it impossible to perform the test

Echocardiography[49]	Evaluation of the heart-lung interaction Measures left and right ventricular function	Same as above Cardiac images are difficult to visualize in some patients

Asynchrony[36, 50]	Quantification of asynchrony and better adjustment of parameters and modes of mechanical ventilation during weaning	Automatic devices that are validated for clinical use are missing

EIT[51–54]	Non-invasive and radiation-free Real-time visualization of aeration and collapsed lung and swings during weaning Evaluation of pulmonary perfusion	Artifacts caused by changes in thoracic shape, providing three-dimensional absolute/relative images with better resolution

Biomarkers[55]	Evaluation of VILI and P-SILI worsening during mechanical ventilation weaning	Influence by extrapulmonary inflammatory response

EIT: Electrical Impedance Tomography; VILI: Ventilator-Induced Lung Injury; P-SILI: patient self-inflicted lung injury.

**Table 2:** Future potential suggestions to evaluation in VM weaning for ARDS.

WEANING IN ARDS
(1) Control of the Illness (reducing inflammation)
(2) PaO2/FiO2>200 and PEEP≤10 cmH2O
(3) Evaluate:
(a) Pulmonary mechanics during the spontaneous ventilation test:
(i) Measure TV e DP – caution TV>8ml/kg and/or DP>13 [56]
(ii) If available – monitoring Pes [42, 57]
(iii) Bedside alternative for the evaluation of pulmonary mechanics: Administration of small doses of sedatives and short-acting NMB (propofol 10mg IV and if necessary succinylcholine 2-4mg IV) and change to VCV to measurements [58]
(b) Asynchrony and Ventilatory Drive:
(i) Asynchrony Index (failure if>10%)[59]
(ii) P0.1 (consider high drive if>3.0)[44, 45]
(c) Imaging Monitoring:
(i) EIT (tidal variation of impedance (TIV), the changes in end-expiratory lung impedance (ΔEELI) – failure if global inhomogeneity index (GI) value>40 [53]
(ii) US (lung score >17 is predictive of postextubation distress [49, 60])
(iii) Echocardiography (qualitative right ventricular failure and diastolic dysfunction)[49]
(4) Management with High TV, DP, Asynchrony Index, P0.1 or worse of regional aeration:
(i) Eliminate stress factors (pain, anxiety and delirium) and sedation adjustment – try dexmedetomidine or propofol. Avoid bolus of fentanyl (can lower RR and increase TV)
(ii) Test increment in PEEP to 12cmH2O
(iii) Alternative ventilatory modes to improve asynchronies – PAV [61] or NAVA [62]
(iv) Patients with refractory weaning: use partial NMB [63] and ECMO [64]

ARDS: acute respiratory distress syndrome; MV: mechanical ventilator; PEEP: positive end-expiratory pressure; TV: tidal volumes; DP: driving pressure; Pes: esophageal pressure; P0.1: pressure 100 ms after the onset of an inspiratory effort; EIT: electrical impedance tomography; VCV: volume control ventilation; US: ultrasound; lPAV: proportional-assist ventilation; NAVA: neurally adjusted ventilator assist; NMB: neuromuscular blockade; ECMO: extracorporeal membrane oxygenation.

## Data Availability

The data used for this review were consulted through the availability of journal access.

## Abbreviations

ARDS: Acute respiratory distress syndrome
MV: Mechanical ventilator
VILI: Ventilator-induced Lung Injury
COPD: Chronic obstructive pulmonary disease
TV: Tidal volumes
PEEP: Positive end-expiratory pressure
RR: Respiratory rate
NMB: Neuromuscular blockade
ECMO: Extracorporeal membrane oxygenation
SBT: Spontaneous breathing trial
PSV: Pressure support ventilation
Ppl: Pleural pressure
P-SILI: Patient self-inflicted lung injury
f/VT: Frequency-to-tidal volume ratio
DP: Driving pressure
Pes: Esophageal pressure
P0.1: Pressure 100 ms after the onset of an inspiratory effort
EIT: Electrical impedance tomography
PCV: Pressure control ventilation
PAV: Proportional-assist ventilation
NAVA: Neurally adjusted ventilator assist
EAdi: Electrical activity of the diaphragm.
